# Analysis of the small RNA *P16/RgsA* in the plant pathogen *Pseudomonas syringae* pv. *tomato* strain DC3000

**DOI:** 10.1099/mic.0.063826-0

**Published:** 2013-02

**Authors:** So Hae Park, Bronwyn G. Butcher, Zoe Anderson, Nola Pellegrini, Zhongmeng Bao, Katherine D’Amico, Melanie J. Filiatrault

**Affiliations:** 1 Department of Plant Pathology and Plant-Microbe Biology, Cornell University, Ithaca, NY 14853, USA; 2 Plant–Microbe Interactions Research Unit, Robert W. Holley Center for Agriculture and Health, Agricultural Research Service, United States Department of Agriculture, Ithaca, NY 14853, USA

## Abstract

Bacteria contain small non-coding RNAs (ncRNAs) that are responsible for altering transcription, translation or mRNA stability. ncRNAs are important because they regulate virulence factors and susceptibility to various stresses. Here, the regulation of a recently described ncRNA of *Pseudomonas syringae* pv. *tomato* DC3000, *P16*, was investigated. We determined that RpoS regulates the expression of *P16*. We found that deletion of *P16* results in increased sensitivity to hydrogen peroxide compared to the wild-type strain, suggesting that *P16* plays a role in the bacteria’s susceptibility to oxidative stress. Additionally the *P16* mutant displayed enhanced resistance to heat stress. Our findings provide new information on the regulation and role of this ncRNA in *P. syringae*.

## Introduction

Small non-coding RNAs (ncRNAs) are transcripts that function in a bacterial cell structurally as RNA molecules rather than as templates for translation into polypeptides (Majdalani *et al.*, 2005). ncRNAs play important regulatory roles in bacterial stress responses to diverse environmental signals, such as changes in temperature, osmolarity, iron and oxidative stress (Gottesman, 2005; Gottesman *et al.*, 2006; Massé *et al.*, 2007; Romby *et al.*, 2006; Silvaggi *et al.*, 2006), and have key roles in the regulation of virulence factors in a variety of pathogens (Altier *et al.*, 2000; Caswell *et al.*, 2012; De Lay & Gottesman, 2012; Hébrard *et al.*, 2012; Kreikemeyer *et al.*, 2001; Le Rhun & Charpentier, 2012; Lenz *et al.*, 2005, 2004; Mangold *et al.*, 2004; Sonnleitner *et al.*, 2012), including *Pseudomonas aeruginosa* (Burrowes *et al.*, 2005; Heurlier *et al.*, 2004). A single ncRNA species can directly regulate multiple genes, leading to pleiotropic effects (Gottesman *et al.*, 2006).

Many ncRNAs require the bacterial chaperone Hfq to perform their regulatory functions. Hfq facilitates the interaction of ncRNAs with mRNA targets (Majdalani *et al.*, 2005; Vogel & Luisi, 2011). Binding of the ncRNA to the mRNA results in an increase or decrease in the stability and/or translation of the mRNA (Vogel & Luisi, 2011). Several genome-wide approaches, including RNomics and deep sequencing, have discovered many of the ncRNA and mRNA targets of Hfq and have shown that this chaperone might affect the expression of up to 20 % of all genes in some bacteria (Chao & Vogel, 2010). Thus it is not surprising that Hfq mutants often display defects in a wide range of cellular processes, including quorum sensing, biofilm formation, stress tolerance and virulence (Chao & Vogel, 2010).

Several ncRNAs were identified using sRNAPredict2 and found to be conserved in the pseudomonads (Livny *et al.*, 2006). One, termed *P16* or *RgsA*, has been further studied in *Pseudomonas aeruginosa* and expression was found to be dependent upon RpoS, the sigma factor primarily responsible for the regulation of genes during stationary phase (González *et al.*, 2008), and indirectly influenced by the global response regulator GacA. In the wild-type strains of *Pseudomonas fluorescens* and *P. aeruginosa*, expression of this ncRNA is almost absent during mid-exponential growth but abundant during the stationary phase. Also, expression was reduced twofold in a GacA mutant compared to the wild-type strain. However, no GacA binding site was identified, so the observed effect was reported to be most likely indirect.

The function of *P16/RgsA* is not known; however, it was reported that deletion of this ncRNA resulted in enhanced sensitivity to hydrogen peroxide in *P. fluorescens* CHA0 (González *et al.*, 2008), suggesting that this ncRNA targets genes involved in resistance to oxidative stress. In this study we investigated the regulation and function of *P16/RgsA* in the plant pathogen *Pseudomonas syringae* pv. *tomato* DC3000.

## Methods

### 

#### Bacterial strains/growth conditions.

*Pseudomonas syringae* pv. *tomato* DC3000 (hereafter referred to as DC3000) was routinely cultured on King’s B (KB) agar (King *et al.*, 1954) or on modified Luria medium (LM) (10 g Bacto tryptone, 6 g yeast extract, 0.6 g NaCl, 0.4 g MgSO_4_.7H_2_O, and 1.5 g K_2_HPO_4_ per litre) (Hanahan, 1983) at 28 °C or at room temperature.

#### Creation of *rpoS* and *P16* mutants in *P. syringae* pv. *tomato* DC3000.

Construction of the DC3000 *rpoS* mutant was carried out by PCR amplification of an internal 800 bp sequence of *rpoS* from the DC3000 genome and cloning into pKnockout-Ω (Windgassen *et al.*, 2000). The resulting plasmid was introduced into DC3000 via electroporation. Since pKnockout cannot replicate in DC3000, single-crossover integrants were selected for resistance to spectinomycin. Orientation of integration was determined by PCR.

A P16 (PSPTO_5560) deletion mutant (Δ*P16*) was created using a pK18mobsacB plasmid (Schäfer *et al.*, 1994). pK18mobsacB/Δ*P16* was created by PCR amplification of DNA fragments of approximately 1.0 kb that flank *P16*. Gel-purified PCR fragments were joined by a second PCR amplification with primers containing *Bam*HI and *Hin*dIII restriction sites. The product was gel purified, digested with *Bam*HI and *Hin*dIII, and cloned into pK18mobsacB digested with the same restriction enzymes. The pK18mobsacB deletion construct was confirmed by sequencing (Cornell University Life Sciences Core Laboratories Center) before introduction into DC3000 via electroporation. Integration events were selected on KB medium containing 50 µg kanamycin ml^−1^ and then transferred to 10 % sucrose medium to select for crossover events that resulted in the loss of the *sacB* gene. Sucrose-resistant colonies were screened by PCR and positive clones (those containing the deletion) were confirmed by sequencing.

#### Evaluating susceptibility to oxidative stress.

Wild-type (WT) DC3000 and Δ*P16* were grown on KB plates for 2 days (King *et al.*, 1954). Overnight cultures were prepared in liquid KB, and incubated at 28 °C with shaking. The next morning, 1 ml of overnight culture was pelleted and resuspended in 3 ml liquid MG (mannitol-glutamate medium; Bronstein *et al.*, 2008). A 30 % hydrogen peroxide solution was added to the culture to a final concentration of 30 mM (Péchy-Tarr *et al.*, 2005). No hydrogen peroxide was added to the control cultures. Cultures were incubated at 28 °C with shaking for 30 min then serially diluted. Dilutions were plated on KB plates and incubated at room temperature until colonies were visible and the number of colonies could be enumerated. Three biological replicates were evaluated. The number of colonies for both the control and the experimental tests was averaged for the three biological replicates. The statistical significance was analysed by using a one-tailed *t*-test for two independent samples with unequal variances.

#### Sensitivity to heat stress.

Sensitivity to heat shock was based on the protocol described by Schurr *et al.* (1995). WT DC3000 and the Δ*P16* and Δ*rpoS* mutants were grown on KB plates for 2 days. Cultures were prepared in liquid KB, and incubated at 28 °C with shaking. The next morning, 1 ml of each overnight culture was centrifuged for 5 min at 15 000 r.p.m., the supernatant was removed, and the cell pellet was resuspended in 3 ml liquid MG. Next, the cultures were serially diluted in fresh liquid MG for the first time point (*t* = 0). Aliquots (100 µl) of the dilutions were spread on KB plates and were incubated until colonies were visible and the number of colonies could be enumerated. The cultures were then incubated at 42 °C. Every 15 min, the cultures were serially diluted in fresh medium and 100 µl aliquots of the dilutions were spread on KB plates. The plates were incubated at room temperature until colonies were visible and the number of colonies could be enumerated.

#### NaCl sensitivity assay.

WT DC3000 and the Δ*rpoS* and Δ*P16* mutants were grown overnight in KB medium at 28 °C with aeration. The next day cells were pelleted, and washed and resuspended in MG supplemented with 1.5 M sodium chloride. Resuspended cells were incubated at 28 °C with aeration, aliquots were taken periodically, and serial dilutions of the samples were plated on KB plates to determine the c.f.u.

#### Creation of reporter constructs.

The putative promoter region for *P16* was amplified via PCR using chromosomal DNA isolated from DC3000. Primers were designed to amplify a region spanning 150 nt upstream of the transcriptional start site and including the first 14 nt of the *P16* gene. The sense primer was designed to have a CACC overhang on the 5′ end to ensure directional cloning into pENTR/D-TOPO vector (Invitrogen). The amplicons were separated by agarose gel electrophoresis. The DNA fragment was extracted from the gel using the Qiagen Gel Extraction kit and cloned into the pENTR vector (pENTR Directional TOPO Cloning kit; Invitrogen). To ensure that the putative *P16* promoter region was successfully cloned into the pENTR vector, the insert was sequenced (Life Sciences Core Laboratories Center at Cornell University).

The promoter region was moved into destination vectors pBS58 and pBS59 (Markel *et al.*, 2011) using Gateways LR Clonase II Enzyme mix (Invitrogen). The Gateway cassette in these plasmids is located upstream from a promoterless *lux* gene. The destination vector pBS58 was designed so that the cloned promoter is in the same orientation as the *lux* gene, while pBS59 was designed as a control with the cloning site reversed so that the promoter is cloned in the opposite orientation to the *lux* gene. Plasmids were transformed into One Shot TOP10 Chemically Competent *Escherichia coli* (Invitrogen).

#### Promoter fusion assay.

pBS58/*P16* promoter and pBS59/*P16* promoter plasmids were introduced into WT DC3000 and the Δ*rpoS* mutant via electroporation. Each strain was grown in KB or LM (Pressler *et al.*, 1988), with appropriate antibiotics for approximately 22 h at 28 °C with shaking. Optical density (OD_600_) of the overnight cultures was measured, and each strain was diluted to an OD_600_ of 0.1 in fresh KB, LM or MG. Aliquots (600 µl) of the culture were dispensed into three 200 µl wells in a 96-well plate for three technical replicates. OD_600_ and relative luminescence were measured immediately for an initial measurement (*t* = 0) with a Tecan GENios microplate reader, using Magellan Data Analysis software. Cultures were shaken at room temperature. Both OD_600_ and relative luminescence were measured at 1 h intervals. Relative luminescence values for each technical replicate were normalized by dividing the raw luminescence value by the OD_600_ (Schagat *et al.*, 2007). Three biological replicates were obtained. Technical replicates were averaged for each biological replicate. Means and standard deviations for each of the biological replicates were calculated. Statistical significance was assessed using a one-way ANOVA test.

#### RNA isolation.

Total RNA was prepared with the RNeasy kit (Qiagen) following the manufacturer’s instructions, using the optional on-column DNase I digestion and with the exception that lysozyme was used at a concentration of 5 mg ml^−1^. RNA was treated twice with DNase I (Ambion) to remove residual DNA and then cleaned and concentrated using the MinElute kit (Qiagen).

#### Quantitative real-time PCR (qRT-PCR).

Total RNA (100 ng) extracted from DC3000 was reverse transcribed in a thermocycler using the qScript cDNA Supermix (Quanta Biosciences) according to the manufacturer’s instructions. qRT-PCR was performed using IQ SYBR green Supermix (Bio-Rad) on an iQ5 multicolor real-time detection system (Bio-Rad). The PCR assay was carried out with one cycle at 95 °C for 2.5 min followed by 35 cycles of 95 °C for 15 s and 60 °C for 30 s. The resulting cycle threshold (*C*_t_) values were calculated by the software and analysed using the relative standard curve method (Vencato *et al.*, 2006). *C*_t_ values of each gene tested were normalized to the *C*_t_ values of the housekeeping gene *gap1* (PSPTO_1287) to obtain relative expression data for each gene.

#### Creation of a strain expressing a FLAG-tagged RpoS.

The *rpoS* coding region was amplified with primers oSWC01564 and oSWC01565 (which contains the FLAG sequence followed by a stop codon; see Table S1, available with the online version of this paper, for sequences of all primers) using the Expand High Fidelity PCR System (Roche). The 1.04 kb PCR product was gel purified using the Zymoclean Gel DNA Recovery kit (Zymo Research) and cloned into pENTR/SD/D (Invitrogen) by directional TOPO cloning (Invitrogen) to create pBB25. The pZA01 expression construct (where *rpoS*-FLAG is expressed under the control of the constitutive *nptII* promoter) was created by an LR reaction with pBS46 (Swingle *et al.*, 2008) using LR Clonase II enzyme mix (Invitrogen). This plasmid was then transformed into WT DC3000 by electroporation, creating strain ZAPS01. As a negative control, the empty vector pBS60 was also transformed into WT DC3000, creating strain ZAPS03. The colonies were analysed by PCR to verify the presence of the *rpoS*-*FLAG*.

#### Chromatin immunoprecipitation (ChIP) of RpoS.

Strains ZAPS01 and ZAPS03 were grown in LM overnight and ChIP performed as described by Butcher *et al.* (2011). Enrichment of *P16* was determined by qRT-PCR as described above, but using 10 ng purified DNA from the lysate or immunoprecipitated (IP) samples as the template. Enrichment was determined relative to regions within the *gap1* gene (primers oSWC00381/oSWC00382).

#### Construction of a strain expressing FLAG-tagged Hfq.

Integration of the FLAG epitope-encoding tag at the 3′ end of *hfq* was achieved using a pK18mobsacB (Schäfer *et al.*, 1994) based construct as follows. A region containing *hfq* and upstream sequence was amplified using oligomers oSWC05086 and oSWC05087. Primer oSWC05087 inserts the *FLAG* sequence in front of the *hfq* stop codon. A downstream region was amplified using primers oSWC05088 and oSWC05089. oSWC05088 contains a sequence complementary to oSWC05087. These fragments were amplified using the Expand High Fidelity PCR System (Roche). The PCR products were gel purified using the Zymoclean Gel DNA Recovery kit (Zymo Research) and were joined using the Expand High Fidelity PCR System (Roche) with primers oSWC05086 and oSWC05089 and the up and down fragments as the PCR template. The product (including the *hfq* gene with a FLAG epitope-encoding sequence at the 3′ end, up and downstream sequences, and flanking *Xba*I sites) was cloned into pK18MobsacB. This suicide plasmid was introduced into DC3000 by electroporation and integration events were selected on modified LM with 50 µg kanamycin ml^−1^. A selected colony was then subjected to counter-selection on 10 % sucrose to select for the loss of the *sacB* gene. Kanamycin-sensitive colonies were then screened for the presence of the FLAG-tag by PCR with primers oSWC05086 and oSWC02103 (a primer specific to the FLAG sequence) using Premix *Taq* (Ex *Taq* version 2.0; Takara) and a correct colony selected for further experiments. The *hfq*-*FLAG* and flanking areas in this strain was sequenced to confirm that no other mutations had been introduced in these regions during strain construction. The presence of the FLAG-tagged Hfq was confirmed by Western analysis (data not shown).

#### Co-immunoprecipitation of RNAs bound to Hfq.

Co-immunoprecipitation of RNAs bound to FLAG-tagged Hfq was performed as described by Berghoff *et al.* (2011) with the following modifications. WT DC3000 and *hfq*-*FLAG* strains were inoculated in KB medium to a starting OD_600_ of 0.02 and grown with shaking at 28 °C for 24 h (OD_600_ of approximately 6–7). The cells were harvested by centrifugation at 5000 ***g*** for 5 min at 4 °C and washed twice with cold 50 ml Tris-buffered saline (TBS). Pellets were resuspended in 2 ml cold CelLytic B Lysis reagent (Sigma) with 20 µl Longlife Lysozyme (G-Biosciences), 1 mM PMSF and 5 µl RNaseOUT (Invitrogen) added. The cells were lysed by sonication (twice for 15 s at 15 % power using a Fisher Scientific 550 Sonic Dismembrator with a microtip). Insoluble material was removed by centrifugation at 16 000 ***g*** for 10 min at 4 °C, then 180 µl supernatant was removed for preparation of total RNA (lysate control). The remaining cleared lysate was mixed with 40 µl anti-FLAG M2 Affinity Gel (Sigma), which had been washed twice with TBS as described by the manufacturer, and incubated for 2 h at 4 °C under rotation. The resin was collected by centrifugation at 8000 ***g*** for 30 s at 4 °C and the supernatant carefully removed and discarded. The resin was resuspended in 500 µl cold CelLytic B Lysis reagent, transferred to a Spin-X centrifuge tube filter (Sigma), and centrifuged for 30 s at 5000 ***g***. The resin was washed another four times with 500 µl cold CelLytic B Lysis reagent and finally resuspended in 200 µl CelLytic B Lysis reagent and transferred to a new tube for RNA isolation (IP sample). Total RNA was isolated from the lysate control or IP samples using TRI reagent (Invitrogen) according to the manufacturer’s instructions. Total RNA samples were treated twice with DNase I (Ambion) to remove residual DNA. RNA was extracted using acid phenol/chloroform and then precipitated with sodium acetate and ethanol using standard protocols. RNA was stored at −80 °C.

One hundred nanograms of total RNA (from the lysate controls) or enriched RNA (from the IP samples) was reverse transcribed to cDNA using qScript cDNA SuperMix (Quanta) as described by the manufacturer. A total of 15 ng cDNA was used to perform real-time PCR as described above. All primers are listed in Supplementary Table S1. Under each condition the *C*_t_ values were normalized to the *C*_t_ values for the housekeeping gene *gap1*. Enrichment was calculated relative to the values in the wild-type (untagged) control as follows: enrichment = 2^^−[(IP_FLAG_−IP^^_WT_)–(LYS^^_FLAG_–LYS^^_WT_)]^. The immunoprecipitation was repeated and the mean enrichment values are presented in Results.

#### Evaluating virulence in *Arabidopsis thaliana* seedlings.

To assess virulence, the *Arabidopsis* seedling flood-inoculation assay was used (Ishiga *et al.*, 2011) with the following modifications. After seeds were sterilized, they were germinated on half-strength MS medium that was solidified with 0.7 % Phytagel (instead of 0.3 %). The seeds were vernalized for 4 days at 4 °C to break the dormancy and then plated. Plates were incubated at 26 °C with a 12 h light/12 h dark photoperiod and 75 % humidity. Seedlings that were 14 days post-germination were used for the virulence assay.

To perform the inoculation, 40 ml bacterial suspension [sterile distilled water containing 0.025 % Silwet L-77 (OSI Specialties)] was dispensed into the Petri dish containing 14-day-old *Arabidopsis* seedlings, and the plates were incubated for 3 min at room temperature to allow bacteria to adhere. The bacterial culture was poured off and then plates containing inoculated plants were sealed with 3M Micropore 2.5 cm surgical tape and incubated at 26 °C with a 12 h light/12 h dark photoperiod and 75 % humidity. To determine the initial number of c.f.u., the inoculum was serially diluted and plated on KB containing 50 µg rifampicin ml^−1^. Rifampicin was used throughout the experiment to ensure that all c.f.u. were DC3000 since the strain is resistant to this antibiotic (Dong *et al.*, 1991).

To determine the bacterial growth in *Arabidopsis* leaves, internal bacterial c.f.u. were determined at 72 h post-inoculation. The bacterial population inside the plants was evaluated from two independent seedlings grown in a single Petri dish. Inoculated seedlings were collected by cutting the hypocotyls in order to separate the plants above the agar from the roots in the Phytagel plate, and the total weight of inoculated seedlings was determined. Next, seedlings were surface-sterilized with 30 % hydrogen peroxide for 5 min. After washing three times with sterile distilled water, each seedling was placed into a single well of a 96-deep-well plate, and 200 µl sterile distilled water and two steel beads were added to the well. The plate was covered using two sealing films to prevent the steel beads from breaking through the film during the grinding process. The seedlings were ground using a 5G Mixer (Fluid Management) for 2.5 min. The solution containing the ground seedlings was serially diluted and plated onto KB plates containing 50 µg rifampicin ml^−1^. C.f.u. were counted after 48 h and normalized to c.f.u. per mg plant material.

#### Tomato dip-inoculation.

Tomato cv. Moneymaker was grown in 16 h light/8 h dark cycles at 25 °C in a greenhouse for 4 weeks. Plants were acclimatized on a bench top in open air for 24 h and were then bagged to create a sealed, high-humidity chamber for 24 h prior to dip inoculation. Prior to infection, bacterial cultures were spread onto KB agar plates and incubated at 27 °C for 24 h. The resulting bacterial lawns were then suspended in 10 mM MgCl_2_ to an OD_600_ of 0.2 (~10^7^ c.f.u. ml^−1^). Tomato plants were infected by gentle agitation for 30 s in 10-fold dilutions of the initial bacterial suspensions made in 1 M MgCl_2_+0.01–0.2 % Silwet L-77. Following bacterial inoculation, the tomato plants were allowed to air dry on the bench top for 1 h, then incubated in a growth chamber at 80 % relative humidity, 25 °C and 16 h light/8 h dark. Tomato leaf tissue samples were harvested using a 4 mm cork borer. Bacteria were extracted from plant tissue samples by shaking them in 0.2 ml fresh 10 mM MgCl_2_ at room temperature with steel beads for 2.5 min. Extracted bacteria were then serially diluted in 96-well plates and spotted on KB containing 50 µg rifampicin ml^−1^. Spots containing >5 and <30 colonies were used to quantify the number of c.f.u. per g leaf tissue. Plants were observed daily for the development of disease symptoms.

## Results

### The ncRNA *P16* is directly regulated by RpoS

**Fig. 1.**
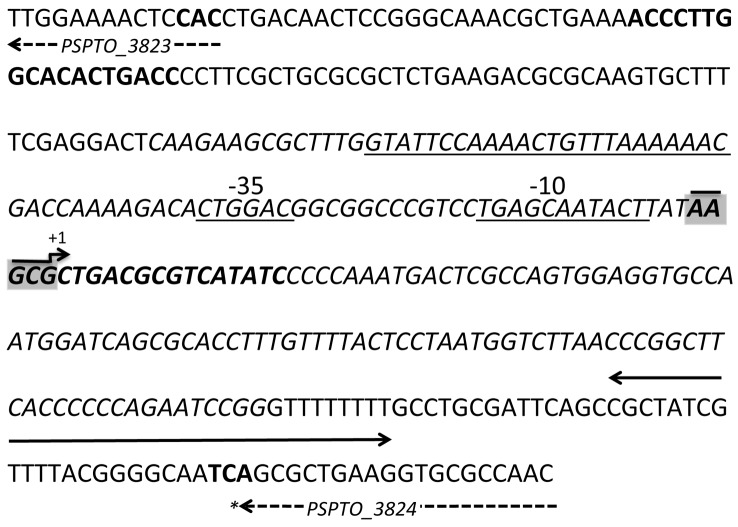
Genomic sequence of *P16* and the putative RpoS promoter sequence. The transcriptional start sites (reported by Filiatrault *et al.*, 2011) are highlighted in grey. The putative RpoS promoter sequence predicted by González *et al.* (2008) is underlined. A predicted terminator sequence is indicated by the solid arrow and the beginning and end of the neighbouring coding sequences are indicated by dashed arrows. The sequence of *P16* predicted in Rfam and annotated as PSPTO_5660 extends upstream of the reported *P16* transcriptional start site and is shown in italics. Primers used to clone the promoter region are shown in bold.

**Fig. 2.**
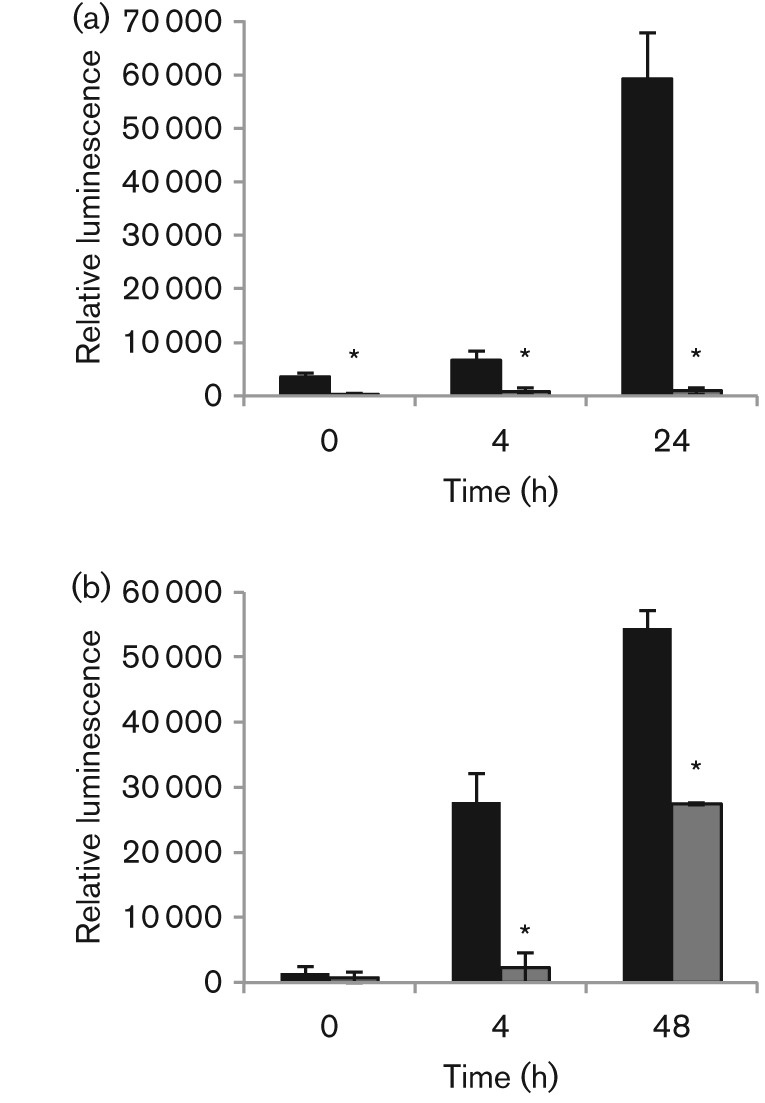
*P16* expression is regulated by RpoS. Expression from a *P16-lux* promoter fusion was compared between WT (black bars) and Δ*rpoS* mutant (striped bars) in KB medium (a) and MG medium (b). As a control the same promoter region was cloned upstream of *lux*, but in the opposite orientation, and expression tested in WT and Δ*rpoS* mutant strains. Relative luminescence values for the controls were <10 000. Relative luminescence is the ratio of luminescence to OD_600_. The mean±sd of three independent experiments is presented. There was a significant difference in relative luminescence between the WT and Δr*poS* strains carrying the *P16-lux* fusion in KB medium (*P*<0.001 at all time points, as denoted by *).

In DC3000, an Rfam prediction for *P16* (PSPTO_5660) is located between PSPTO_3823 and PSPTO_3824 (Filiatrault *et al.*, 2010) [Rfam: http://rfam.sanger.ac.uk]. Our group reported transcriptional activity for this region when cells were grown in MG medium under low-iron conditions (Filiatrault *et al.*, 2010). However, we have since noticed a discrepancy in length between the *P16* family reported in Rfam and the reported *P16/RgsA* (González *et al.*, 2008) (Fig. 1). The predicted coordinates reported by Rfam appear to contain an additional 87 nt upstream of the transcriptional start site and the sequence contains the reported RpoS promoter region (González *et al.*, 2008). Since González *et al.* (2008) reported an RpoS binding site located upstream of *P16* in DC3000 (Fig. 1) we investigated if RpoS regulates expression of *P16* in DC3000. Promoter fusion constructs were introduced into WT DC3000 and the Δ*rpoS* mutant strain. Fig. 2 shows that there was a significant reduction in the relative luminescence of the *lux* reporter in the Δ*rpoS* mutant compared to the WT strain in two media (KB and MG). However, when cells were grown in MG, the expression was not completely abolished and some expression for *P16* was detected in the Δ*rpoS* mutant. The results also indicate that *P16* is expressed in both media, with expression increasing throughout growth and highest expression occurring during stationary phase.

The data from the *lux* reporter assay indicate the activity of the *P16* promoter. To determine the abundance of *P16* transcript, qRT-PCR was performed between the WT strain and the Δ*rpoS* mutant. The Δ*rpoS* mutant showed an eightfold reduction in the level of *P16* transcript compared to the WT in KB medium and an 18-fold reduction in the level of *P16* transcript compared to the WT in MG medium (Fig. S1). Taken together, these data indicate that RpoS regulates expression of *P16*. The regulation of *P16* by RpoS could be direct or indirect but the presence of a putative RpoS binding site suggests direct regulation. ChIP was used to detect the binding of RpoS to the upstream region of *P16*. A 1.8–5-fold enrichment of *P16* over the *gap1* gene was observed (Fig. S2). These results show that RpoS directly regulates the expression of *P16*.

### *P16* influences the response of *P. syringae* DC3000 to diverse environmental conditions

**Fig. 3.**
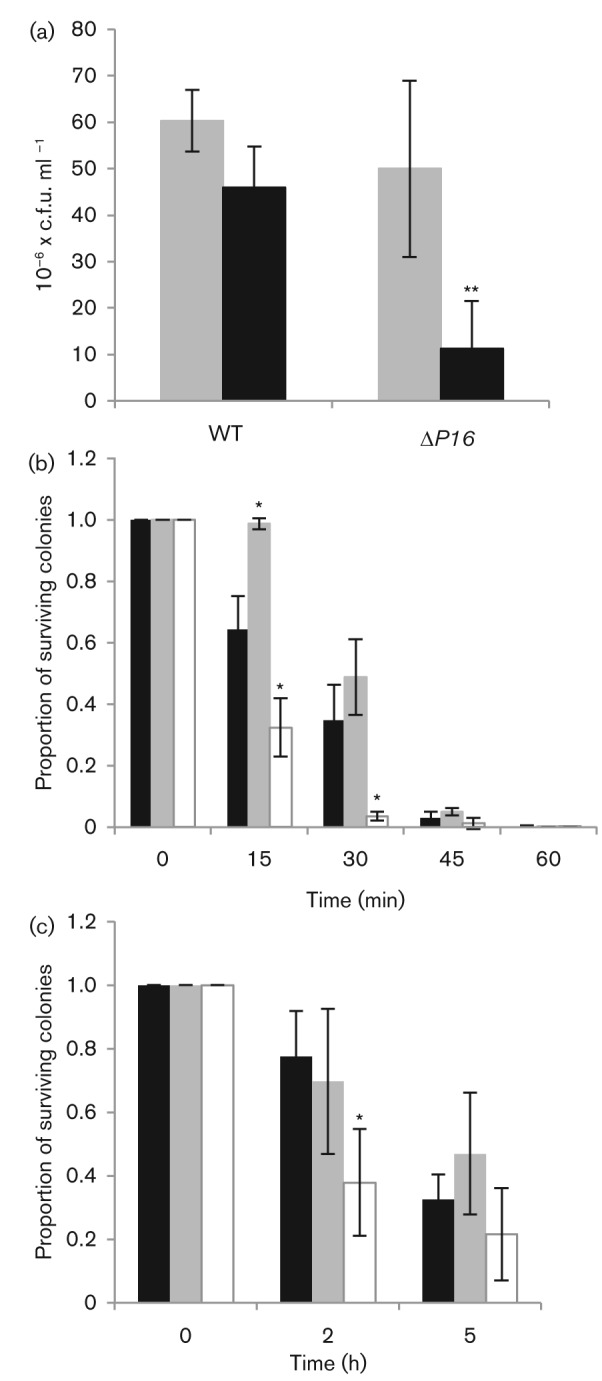
*P16* is involved in response to various stresses. (a) *P16* is involved in the response to hydrogen peroxide. The numbers of c.f.u. were compared between the WT and the Δ*P16* mutant without (grey bars) and with (black bars) the addition of hydrogen peroxide. There was a significant reduction in c.f.u. (more than 50 %) when hydrogen peroxide was added to the Δ*P16* mutant cells (*P*<0.01, as denoted by **). (b) *P16* influences susceptibility to heat shock. The proportion of surviving cells after incubation at 42 °C was compared between WT (black bars), Δ*P16* mutant (grey bars) and Δ*rpoS* mutant (white bars) cells. Significant difference was observed compared to WT (*P*<0.01, as denoted by *). (c) *P16* does not play a role in the response to salt stress. The proportion of surviving cells after addition of 1.5 M NaCl was compared between WT (black bars), Δ*P16* mutant (grey bars) and Δ*rpoS* mutant (white bars) cells. Significant difference was observed compared to WT (*P*<0.01, as denoted by *).

RpoS has also been shown to play a role in protecting bacterial cells from oxidative stress (González *et al.*, 2008), heat stress (Jørgensen *et al.*, 1999; Suh *et al.*, 1999) and osmotic stress (Kidambi *et al.*, 1995). Therefore, we evaluated the role of *P16* in the response of DC3000 to these environmental factors. Susceptibility to oxidative stress was analysed by an assay in which the bacteria were exposed to hydrogen peroxide. Fig. 3(a) shows that the addition of 30 mM hydrogen peroxide to Δ*P16* cultures resulted in a significant reduction (>50 %) in the number of colonies (*P*<0.01). Based on these data, it is likely that *P16* plays a role in protecting the bacteria from oxidative stress.

As shown in Fig. 3(b), the proportion of surviving cells for the Δ*P16* mutant remained unchanged after 15 min exposure to heat stress, whereas the WT and Δ*rpoS* mutant demonstrated a significant reduction in the proportion of surviving cells when exposed to heat shock at any of the time points tested. In addition, the Δ*rpoS* mutant showed a significant reduction in the proportion of surviving cells at *t* = 15 min and *t* = 30 min compared to the WT. Therefore, it is likely that *P16* participates in the tolerance to heat stress.

No difference in growth in 1.5 M sodium chloride was observed between WT and Δ*P16* mutant, while the Δ*rpoS* mutant displayed a significant reduction in the number of colonies (Fig. 3c). This suggests that it is unlikely *P16* plays a role in protecting the bacteria from osmotic stress.

### *P16* does not influence virulence

**Fig. 4.**
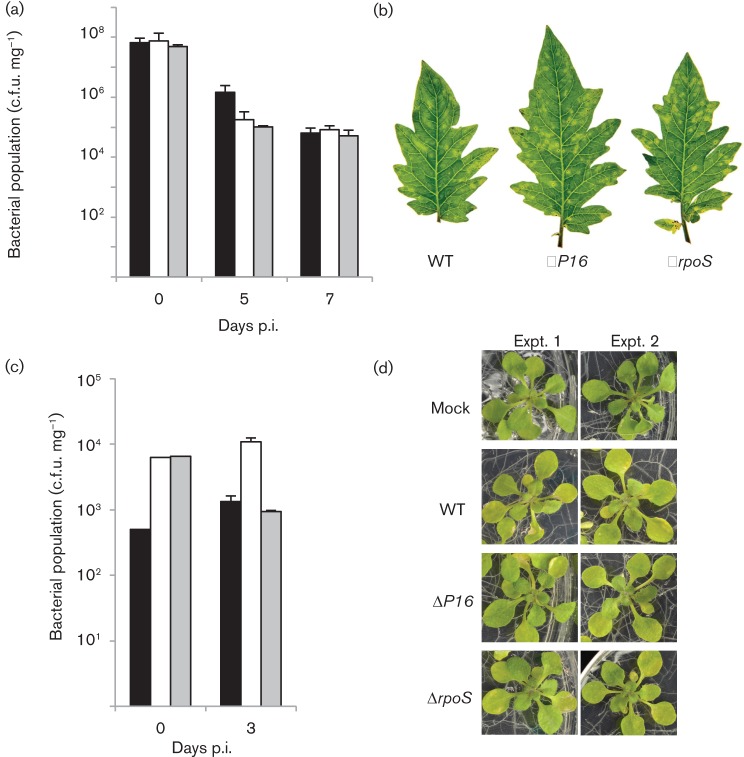
*P16* does not influence virulence. (a) Four-week-old tomato cv. Moneymaker tomato plants were dipped in suspensions containing ~10^7^ c.f.u. ml^−1^ of either WT, Δ*P16* or Δ*rpoS*. At the time points indicated below the graph, bacteria were extracted from leaves and plated on KB containing rifampicin for enumeration. The values plotted are the means±sd obtained from technical replicates. Similar results were obtained in two repetitions of the experiment. (b) Tomato leaves infected in (a) were photographed at 5 days post-infection (p.i.). Similar results were obtained in a repeated experiment. (c) Bacterial populations of WT, Δ*P16* or Δ*rpoS* in *Arabidopsis*. Bacterial populations were quantified at 0 and 3 days p.i. Vertical bars indicate the standard deviations for two independent experiments. (d) Disease phenotype of *Arabidopsis* seedlings flood-inoculated with a bacterial suspension of WT, Δ*P16* or Δ*rpoS*. Mock-inoculated seedlings were flooded with sterile distilled H_2_O containing 0.025 % Silwet L-77. Photographs were taken 3 days p.i.

Since exposure of bacteria to oxidative stress is an important part of the plant defence response, we investigated whether *P16* contributes to the growth and virulence of DC3000. Tomato plants were dipped in suspensions of the WT, the Δ*P16* mutant and the Δ*rpoS* mutant. Although the Δ*P16* mutant and the Δ*rpoS* mutant appeared to be slightly less abundant than the WT at 5 days post-inoculation, by day 7 all the strains produced approximately the same number of colonies (Fig. 4a). All of the inoculated plants developed lesions and no differences were observed among the lesions produced by the three strains at day 5 (Fig. 4b). These data suggest that *P16* and *rpoS* may be critical for growth in early stages in infection in tomato plants.

We additionally examined growth and virulence of the WT, the Δ*P16* mutant and the Δ*rpoS* mutant strains in *Arabidopsis* seedlings. At 3 days post-inoculation, the Δ*P16* mutant and the Δ*rpoS* mutant were as efficient at growing *in planta* as the WT strain (Fig. 4c). In addition, *Arabidopsis* seedlings infected with the WT, the Δ*P16* mutant and the Δ*rpoS* mutant displayed the same necrotic symptoms (Fig. 4d). Based on these data, it is unlikely that *P16* plays a role in virulence in DC3000.

### *P16* interacts with Hfq

**Fig. 5.**
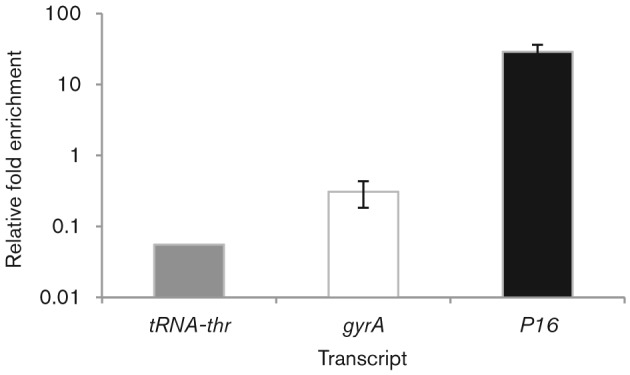
*P16* interacts with Hfq. RNA was isolated from WT and Hfq-FLAG-tagged strains before (lysates) or after immunoprecipitation using anti-FLAG M2 Affinity Gel. RNA was reverse transcribed and used to perform qRT-PCR for *P16*, *tRNA-thr2* and *gap1* transcripts. *C*_t_ values were normalized to *gap1*. The results show enrichment of *P16* transcript, but not the transcripts of the housekeeping gene *gyrA*, or the tRNA *tRNA-thr2*.

Many bacterial ncRNAs use the chaperone Hfq to facilitate the interaction of ncRNAs with target mRNAs (Majdalani *et al.*, 2005; Vogel & Luisi, 2011). Hfq is an RNA-binding protein that controls a number of different cellular processes and is highly conserved in a wide variety of bacteria, including all completely sequenced pseudomonads. Mutations in *hfq* attenuate virulence in several pathogenic bacteria (Berghoff *et al.*, 2011; Christiansen *et al.*, 2004; Ding *et al.*, 2004; Geng *et al.*, 2009; Liu *et al.*, 2010; Sonnleitner *et al.*, 2003; Torres-Quesada *et al.*, 2010). In DC3000, we are unable to investigate phenotypes associated with the loss of *hfq* because we are not able to construct an *hfq* mutant (the loss of *hfq* in DC3000 is probably lethal). To further characterize *P16* we expressed an epitope-tagged version of Hfq and determined if *P16* was able to interact with Hfq. We found that *P16* is enriched following RNA immunoprecipitation of strains containing a FLAG-tagged Hfq (Fig. 5).

## Discussion

The stationary-phase sigma factor RpoS (σ^S^) has been shown to be important for optimal stress response in *Pseudomonas*. *rpoS* mutants of *Pseudomonas* frequently display reduced survival in stationary phase when exposed to environmental stresses, such as UV radiation, desiccation, heat and osmotic stress. For example, RpoS is important for survival of *P. aeruginosa* under osmotic shock, heat shock and oxidative stress conditions (Jørgensen *et al.*, 1999; Suh *et al.*, 1999). RpoS has been shown to contribute to tolerance to stresses such as oxidative stress in *P. fluorescens* (Heeb *et al.*, 2005; Stockwell *et al.*, 2009). Although differences in the response to various stresses and fitness has been observed between various strains (Hagen *et al.*, 2009; Stockwell & Loper, 2005; Stockwell *et al.*, 2009), very few studies have evaluated the role of RpoS in the plant pathogen *P. syringae*. The *rpoS* gene in *P. syringae* pv. *syringae* B728a is important in surviving exposure to the near-UV in sunlight (Miller *et al.*, 2001), but no studies have reported its role in response to other environmental stresses. Here we report that an RpoS mutant of *P. syringae* DC3000 is sensitive to heat stress, but is not altered in virulence. As noted above, specific environmental conditions influence the outcomes that have been observed with RpoS mutants. Therefore, it is possible that RpoS could play a significant role in *P. syringae* when other environmental situations are encountered.

The data presented here show that the transcription of *P16/RgsA* is regulated by RpoS; this is consistent with reports in other pseudomonads. GacA also controls expression of *rpoS* in DC3000 (Chatterjee *et al.*, 2003). Therefore it is likely that *P16/RgsA* in DC3000 is also indirectly controlled by this two-component system.

Oxidative stress plays an important role in the plant environment. Oxidative stress can be caused by a number of reactive oxygen species (ROS), such as superoxide anion, hydrogen peroxide (H_2_O_2_) and hydroxyl radical. ROS are involved in many biological processes. ROS can be produced as a result of normal aerobic metabolism, but also serve as a mechanism to reduce the viability of invading pathogens (Bolwell, 1999). Generation of ROS occurs as part of the defence mechanism in plants against invading pathogens (Bolwell, 1999). In fact, it is recognized that upon infection of plants with DC3000, there is activation of the plant defence responses and as a result there is increased production of ROS (Alvarez *et al.*, 1998; Levine *et al.*, 1994). We observed that the Δ*P16* mutant was more sensitive to hydrogen peroxide than the WT strain. This suggests that *P16* may play a role in resistance to plant defence mechanisms during infection. However, deletion of *P16* did not affect the ability of the pathogen to infect *Arabidopsis* or tomato. It is possible that the amount of hydrogen peroxide produced during this particular infection model is not sufficient to detect a difference in viability or that the number of cells used in our experiments is too overwhelming to observe an effect. Interestingly, Miguel *et al.* (2000) reported that an *oxyR* mutant in *Erwinia chyrsanthemi* is more sensitive to hydrogen peroxide *in vitro*, but this mutant retains full virulence. The data reported by Miguel *et al.* (2000) suggest that there is no direct antimicrobial effect of hydrogen peroxide in plant defence against *E.* *chyrsanthemi* and raise the question as to whether this phenomenon occurs in other plant pathogens. Our data show that this phenomenon occurs in *P. syringae* DC3000 as well. As noted by Miguel *et al.* (2000), more experiments are needed to determine the mechanism involved. It is however still possible that *P16* plays a role in resistance to other ROS present during infection.

Another possible explanation for the lack of a role for *P16* during infection is that stationary phase may not be reached in the plant infection or that RpoS is not needed for *P. syringae* under the conditions tested and other sigma factors known to play a role in the stress response of *P. syringae*, such as AlgU and RpoN, are used in the plant infection when exposed to various environmental stresses. One way to determine if stationary phase is reached in the plant is to determine the expression of genes known to be controlled by RpoS. Currently we do not have any evidence that *P16* is expressed during infection or *in planta*. Studies are under way in our lab to examine the global transcript profile of *P. syringae* during infection.

Temperature is recognized as an important environmental signal and is known to influence production of virulence factors in a number of pathogens. Little is known about how temperature affects the virulence of plant-pathogenic bacteria, though temperature has been shown to influence the pathogenicity of *P. syringae* pv. *glycinea* (Palmer & Bender, 1993), coronatine production (Palmer & Bender, 1993), and the expression of antibiotic compounds and hydrogen cyanide in *P. fluorescens* CHA0 (Humair *et al.*, 2009). Our results are consistent with those reported for RpoS in other pseudomonads in that deletion of *rpoS* results in increased sensitivity to elevated temperatures. Surprisingly, deletion of *P16* resulted in a slight increase in resistance to exposure to higher temperatures. A possible explanation for this discrepancy is that *P16* may target an mRNA that decreases the stability of RpoS. If this were the case then it might be expected that deletion of P16 would result in an increase in resistance to heat stress. It is known that RpoS is regulated at the transcriptional, translational and post-translational level, with different stresses acting at different levels (Battesti *et al.*, 2011), and this would be consistent with our findings. Even so, further experiments will be required to dissect these pathways.

It is possible that *P16* is transcribed at low levels in the Δ*rpoS* mutant and this residual expression results in the observed phenotype. In fact, our data show that *P16* expression is not completely abolished in a Δ*rpoS* mutant background (Fig. 2). This is not uncommon for genes regulated by RpoS. Some genes regulated by RpoS are only induced specifically by RpoS under particularly stressful conditions, whereas others are expressed constitutively by the housekeeping sigma factor RpoD and then expression becomes boosted by RpoS (Battesti *et al.*, 2011). Also, some RpoS-regulated genes require additional transcriptional activators for their expression (Battesti *et al.*, 2011). González *et al.* (2008) noted the presence of a regulatory sequence upstream of *P16* that appears to be conserved in the pseudomonads. The role of this sequence in the regulation of *P16* has yet to be investigated, but it could explain the various responses to different stresses we observed.

Because different responses were observed for *P16* when exposed to oxidative stress and heat stress, it is possible that *P16* may be regulated by several different mechanisms (as noted above). Alternatively, different mRNA targets may be involved in the response to these particular stresses and *P16* may regulate these targets in different ways. mRNA targets have not been reported for the *Pseudomonas* ncRNA *P16*. To identify possible targets for this ncRNA, the DC3000 genome was scanned for target of *P16* using the program IntaRNA (http://rna.informatik.uni-freiburg.de:8080/v1/IntaRNA.jsp). Interestingly, a candidate from this analysis was PSPTO_5535. The interaction between *P16* and PSPTO_5535 is predicted to occur from positions −17 to 22 on PSPTO_5535 and 30–75 on *P16*. PSPTO_5535 is annotated as a hypothetical protein, with an SPFH domain (http://www.pseudomonas.com/). The SPFH superfamily of proteins contain ‘SPFH’ domains named after the proteins stomatin, prohibitin, flotillin and HflK/C (Browman *et al.*, 2007; López & Kolter, 2010). While these proteins are commonly distributed in bacteria, their functions in these organisms are unclear. However, there are reports that they may be involved in stress responses such as those to high salt and antibiotic treatment (Butcher & Helmann, 2006).

We believe this is the first study of *P16* in the plant pathogen *P. syringae* DC3000. Studies are under way to confirm the direct binding of *P16* to PSPTO_5535 and perform a more detailed investigation of the role of *P16* in the tolerance to heat shock.
